# Lipid-Based Intelligent Vehicle Capabilitized with Physical and Physiological Activation

**DOI:** 10.34133/2022/9808429

**Published:** 2022-10-31

**Authors:** Fuxue Zhang, Bozhang Xia, Jiabei Sun, Yufei Wang, Jinjin Wang, Fengfei Xu, Junge Chen, Mei Lu, Xin Yao, Peter Timashev, Yuanyuan Zhang, Meiwan Chen, Jing Che, Fangzhou Li, Xing-Jie Liang

**Affiliations:** ^1^CAS Key Laboratory for Biomedical Effects of Nanomaterials and Nanosafety, CAS Center for Excellence in Nanoscience, National Center for Nanoscience and Technology of China, No. 11, First North Road, Zhongguancun, Beijing 100190, China; ^2^Sino-Danish Center for Education and Research, Sino-Danish College of University of Chinese Academy of Sciences, Beijing 100049, China; ^3^School of Nanoscience and Technology, University of Chinese Academy of Sciences, Beijing 100049, China; ^4^China National Institutes for Food and Drug Control, Beijing 102629, China; ^5^Beijing Advanced Innovation Center for Biomedical Engineering, School of Engineering Medicine, Beihang University, Beijing 100083, China; ^6^Advanced Research Institute of Multidisciplinary Science, School of Life Science, School of Medical Technology (Institute of Engineering Medicine), Key Laboratory of Molecular Medicine and Biotherapy, Key Laboratory of Medical Molecule Science and Pharmaceutics Engineering, Beijing Institute of Technology, Beijing 100081, China; ^7^School of Chemical Sciences, University of Chinese Academy of Sciences, Beijing 100049, China; ^8^Laboratory of Clinical Smart Nanotechnologies, Institute for Regenerative Medicine, Sechenov University, Moscow, Russia; ^9^State Key Laboratory of Quality Research in Chinese Medicine, Institute of Chinese Medical Sciences, University of Macau, Macau SAR, China

## Abstract

Intelligent drug delivery system based on “stimulus-response” mode emerging a promising perspective in next generation lipid-based nanoparticle. Here, we classify signal sources into physical and physiological stimulation according to their origin. The physical signals include temperature, ultrasound, and electromagnetic wave, while physiological signals involve pH, redox condition, and associated proteins. We first summarize external physical response from three main points about efficiency, particle state, and on-demand release. Afterwards, we describe how to design drug delivery using the physiological environment in vivo and present different current application methods. Lastly, we draw a vision of possible future development.

## 1. Introduction

Drug delivery system (DDS) is a progressive pharmaceutical formulation that delivers drugs to specific sites and release in a desired manner and rate. A few decades ago, small molecule drugs were the predominant therapeutic agents and their delivery depended heavily on the physicochemical properties of their own structure. In contrast, with the development of pharmaceutical sciences, the content of drugs has been extended to nucleic acids [1], peptides [2], proteins [3], and even cells [4], which are difficult to apply directly in clinical therapy due to the instability of their physicochemical properties. In this context, the role of DDS is increasingly significant, not only limited to improving the solubility of small molecule drugs but also in controlling the release behavior [5], maintaining the activity of drugs [6], overcoming biological barriers [7], and improving pharmacokinetics [8]. Lipid nanoparticle (LNP) is currently the most widely used and mature DDS in clinical practice. Whether it is more than twenty small molecule DDS formulations that have been marketed or the recent mRNA vaccines for COVID-19, LNP plays a key role in the development of advanced pharmaceuticals [9]. The concept of LNP can be divided into general LNP and special LNP. General LNP is a new DDS that use biocompatible phospholipid materials as carriers to wrap bioactive substances in nuclei or attach them to the surface of nanoparticles. General LNP includes solid lipid nanoparticles, niosomes, transfersomes, nanostructured lipid carriers, micelles, etc., of which liposome is the most representative [10]. And special LNP is described in more articles now as a specific type of particle used to deliver nucleic acids with the development of COVID-19 vaccines [10, 11]. Special LNP is a composite vesicular structure composed of four components: PEGylated lipids, ionizable lipids, structural phospholipids, and cholesterol. Here, we refer to LNPs, collectively, as lipid-based nanoparticles to avoid confusion. Most medical lipid-based nanoparticles have made progress in improving the solubility of small molecule drugs, maintaining the biological activity of large molecule drugs and improving the pharmacokinetics. However, the precise control of drug release behavior is a critical issue for the next generation of lipid-based nanoparticles toward clinical application, which requires the realization of selective drug delivery with timing, quantification, and localization to improve therapeutic efficiency while reducing side effects. In another word, intelligent drug delivery system needs to be summarized to promote rational clinical drug use.

The intelligent DDS contains the following two controllability aspects: (1) intelligent DDS has spatial controllability. It is highly selective in releasing drugs at target sites through disease-specific condition or inflicting signals at lesions directionally; (2) intelligent DDS has time controllability. A stimulus can be applied at a certain time to control the moment of drug release. Different controllability aspect is emphasized based on the type of disease and treatment purpose. Most solid tumors, for instance, were focused on targeting ability [12] . While for glycemic control of diabetes type 1, the drug carrier is required to release insulin on time when postprandial blood glucose rises but stop working after blood glucose reverting to normal [13]. Thus, the intelligent DDS is a drug delivery system that combines sensing, processing, and execution by functional materials, which aims to deliver a drug to lesion or realize the on/off control of drug release according to stimulus signals, in another word, response.

**Figure 1:**
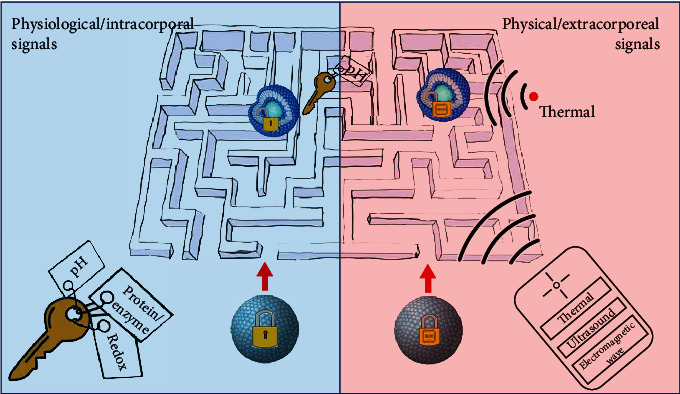
Scheme diagram of intelligent lipid-based nanoparticle. Physical signals, thermal, ultrasound, and electromagnetic wave, act as external remote controls initiate carrier response in circulatory labyrinth. While physiological signals, pH, redox and proteins, act as internal keys induce carrier activation.

“Stimulus-response” mode is an important strategy utilized in materials to achieve intelligent DDS [14]. The mode is in analogue to relationship between lock and key. When lipid-based nanoparticles enter the organism, they are dispersed in a “labyrinth” of circulatory system. Generally, these lipid carrier materials have self-feedback capabilities. Some components can interact with specific stimuli then affects carrier properties, for instance, phase, shape, surface energy, and reaction rate. These natures can be designed to regulate the drug release rate, circulation time, and targeting in many approaches including but not limited to changing conformation [15], effecting interaction between drug and carrier [16], disrupting the structural integrity [17], and removing or degrading the gating molecules [18]. Here, we classify signals sources into extracorporeal and intracorporal stimulation based on their origin. Extracorporeal signals usually act as physical stimulus to cause response of material. It is accessible to change physicochemical properties of the particle by an external “remoter control” such as heat, ultrasound, and electromagnetic waves, while some intracorporal signals can be used to drive drug release. The specific physiological environment, such as pH, redox environment, certain enzyme, or protein, plays a role as key at lesion to open the lock of the carrier. Next, we will summarize the intelligent lipid-based nanoparticles stimulated by extracorporeal physical signals and intracorporal physiological signal in recent years and analyze the Functional lipid components combining with disease characteristics (Figure 1).

## 2. Intelligent Lipid-Based Vehicle Responded with Physical Activation

**Figure 2:**
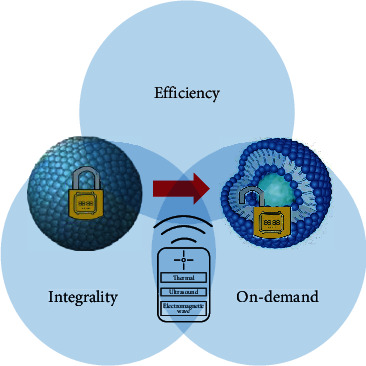
Three main applications of physical signal-activated lipid-based nanoparticles. Efficiency is associated with complete contents release. Integrality is related to preleakage and elimination. On-demand release correlates about precise control quantitatively.

Generally, extracorporeal signals are physical quantities that can be easily controlled by human beings including temperature, ultrasound, electromagnetic wave, and so on. During process of carrier designing, some physical signals directly cause response of carrier through some functional lipids and sensors, while some else need to be converted into other energy and then induce the carrier changing. Usually, adulterants can transform the first signal into chemicals or other energy forms that are easy to induce reaction of lipid components, so as to cause drug release. For instance, light, alternating magnetic fields and ultrasound can be converted into thermal energy. These physical quantities have wide applications, which can be used as a remoter to control carrier behavior. (1) Enhance release efficiency. These signals stimulate physical or chemical changes in the substrate, causing efficient release of the contents at any time of medical procedure; (2) Regulate carrier state. Cyclic state of carrier can be stabilized by “stimulus-response” regulation or response molecule optimization, which allows the cargo to reach destination intact without drug leakage or elimination before targeted sites to avoid toxic side effects; (3) Realize on-demand response. Physical signals can be applied or removed at any time and precisely adjusted strength according to corresponding parameters, which is accessible for multiple release. And efficiency, carrier integrality, and on-demand release are three point that should be concerned about. Efficiency is associated with complete disruption of structure to lead a competent release. Particle integrality is related to avoid side effect caused by preleakage and stable circulation sate. On-demand release correlates about precise control quantitatively rather than a burst release mode. Lipid-based nanoparticles that respond to extracorporeal signal sources will be introduced as follows (Figure 2).

### 2.1. Thermal Response for Lipid Phase Control

Phase transition temperature (T_m_) is an important parameter for liposomes. T_m_ is a temperature at which the liposome bilayer changes from a gel phase to a liquid crystal phase. It is ordered for lipid hydrophobic alkyl chain in gel state, while the chain is disordered in liquid crystal state [19]. During the transition between the two phases, at T_m_, the permeability of the lipid bilayer reaches the summit. Temperature-sensitive liposomes (TSL) based on mentioned principle have been widely reported. 1,2-dipalmitoyl-sn-glycero-3-phosphocholine (DPPC) and hydrogenated soy *sn* -glycero-3 -phosphocholine (HSPC) are two commonly lipid materials to prepare TSL. The ratio between DPPC and HSPC can be used to adjust T_m_ for traditional TSL [20]. However, it has a long way to go for traditional TSL about effectiveness, stability, and precise control.

**Figure 3:**
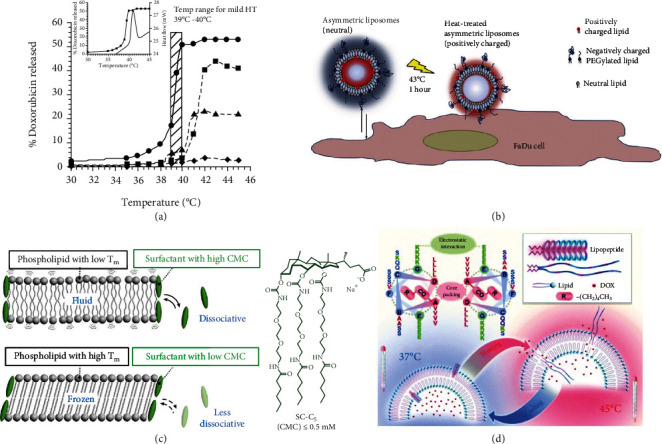
Thermal response for lipid phase control. (a) Nearly 80% of DOX releases in 20 seconds under 42°C in TSL contained MSPC [23]; (b) asymmetric liposomes with different components of inward and outward membrane enhance endocytosis after thermal stimulation [25]; (c) edge stabilizer SC-C5 gives bicelles dilution tolerance and thermoresponsiveness since it has extremely low CMC [38]; (d) thermosensitive on/off switch liposome achieves a reversible drug release on demand [40].

Firstly, effective release of loading drug is a major issue to be addressed. Duke university has developed a lysolipid TSL with doxorubicin (DOX) loading, ThermoDox, consisted by DPPC, 1-palmitoyl-2- hydroxy- sn -glycero-3-phosphocholine (MSPC) and 1,2-distearoyl-sn-glycero-3-phosphoethanolamine-N-polyethylene glycol 2000 (DSPE-PEG2000) [21]. MSPC is a representative around lysolipids. The structure of lysolipid is noncylindrical, which makes molecular head prone to aggregate and form micelles when approaching to T_m_ [22]. TSL mixed with MSPC obtain more stable membrane pores to ensure effective drug release at fluid phase, which improves drug release compared with normal liposomes [23]. The unique property can reduce barrier function of bilayer and increase drug release rate. Nearly 80% of DOX could be released from the TSL in 20 seconds under 42°C (Figure 3(a)). Combination of radiofrequency ablation (RFA) and ThermoDox has been put into Phase III clinical trial in 2008. Intricately, initial progression-free-survival (PFS) data analysis exhibited failed in effectiveness based on total 701 patients, while 285 of whom showed a significant improvement in PFS and overall survival through subsequent retrospective analysis [24]. This statistical difference based on sample population is related to protocol of the trial, including ablation time and primary endpoint selection. And optimized clinical trial has been carried out. Jing et al. developed an asymmetric liposome to enhance drug intracellular release based on thermal triggered endocytosis [25]. The so-called asymmetric liposomes are lipid bilayers with different components of inward and outward membrane (Figure 3(b)). They first prepared symmetric liposome with DPPC, DSPE-PEG 2000, 1,2-diheptadecanoyl-sn-glycero-3-phosphocholine (DHPC), and cationic lipid MVL 5, then modified MVL 5 at outward surface by methyl-terminated polyethylene glycol N-Hydroxysuccinimide ester (PEG-NHS) to obtain asymmetric liposome. MVL 5 is a new multivalent cationic lipid for siRNA delivery, primary amines of which can react with NHS. Thus, the asymmetric liposome with a neutral outer surface contains cationic lipids only in the inner layer. As temperature increased to 43°C, phase transition induces diffusion of cationic lipids from the inner membrane to the outer membrane. The reactivation of cationic lipid on outer surface not only enhances uptake by cells but also promotes contents release inside the cell in a temperature-dependent manner. But animal experiments need to be supplemented since environment is much more complex in vivo.

In addition to iterative optimization of lipid molecules, effectiveness enhancement is also accessible by increasing thermal conversion rate. Inorganic metal nanoparticle, gold, silver [26], and platinum [27], as thermal conversion agent allows a greater concentration of generated local heat, thus increasing response efficacy of carriers, and on the other hand, decreasing thermal damage for normal tissues adjacent to lesion location. Noble metal nanoparticles exposure in the light has enhanced photothermal effects since resonant oscillation of their free electrons, which also known as localized surface plasmonic resonance (LSPR) [28]. Gold nanoparticles (AuNP) as a photothermal enhancer have been widely reported on biomaterial field raised from their stability and biocompatibility. Wang et al. constructed an efficient CRISPR-Cas9 delivery system by condensing plasmids on TAT peptide-modified AuNP and encapsulating the obtained particle with 1, 2-dioleoyl-3-trimethylammoniumpropane (DOTAP), dioleoyl-phosphatidylethanol-amine (DOPE), cholesterol, and DSPE- PEG2000 [29]. The lipid shell compromised due to photothermal effects of AuNP after 514 nm (24 mW cm^−2^) laser irradiation, which induces release of the designed plasmid at cytoplasm. But irradiation band of gold nanospheres is around 510 nm, the limited penetration of which restricts application in deep tissues. Zhan et al. prepared liposomes with DPPC, 1,2-dipalmitoyl-sn-glycero-3-phospho-(1′-rac-glycerol) (DPPG), HS-PEG-DSPE anchored gold nanorods (GNR) for on-demand release of tetrodotoxin and dexmedetomidine in deep tissues [30] . Because of high penetration of near infrared (NIR), GNR photothermal effect induced the release of analgesic compounds from liposomal at rat footpads under 808 nm irradiation. Organic photothermal conversion agents were also developed in lipid-based nanoparticle thermal response such as indocyanine green [31], cyanine [32], and aza-BODIPY [33]. Superparamagnetic iron oxide nanoparticles (SPION) include magnetite (Fe_3_O_4_), maghemite (*γ*-Fe_2_O_3_), and mixed ferrites (MFe_2_O_4_ where M = Co, Mn, Ni, or Zn). They have magnetothermal effect under alternating magnetic field contributed to Brownian and Néel relaxation processes [34]. Tapeinos et al. reported a lipid-based nanovector with superparamagnetic iron oxide nanoparticles inside to against glioblastoma multiforme [35]. The SPION increased temperature 37°C to 43°C by stimulation of alternating magnetic field (AMF), and sustained release of temozolomide resulting into apoptosis and in the subsequent death of glioblastoma cells. Another work was reported by Guo et al. who combined AMF and local precise NIR in single liposome to realize 4.2-fold heating effect amplification [36]. It is a significantly release of DOX at tumor region for the multiple functional liposomes in dual-mode stimulation. The liposomes consist of cholesterol, DPPC, octadecanamine (SA), 1,2-diacyl-SN-glycero-3-phosphoethanolamine-N-[methoxy(poly(ethyleneglycol))-2000] (DSPE-MPEG), and DSPE-PEG2000-methotrexate.

Secondly, integrality is another problem that needs to be solved since preleakage of drugs at body temperature. Especially for high activity drugs, it is hazardous for preleakage prior to target site even at trace amounts level. Yin et al. synthesized a phase-change material (PCM) loaded with both thrombin (Thr) and photothermal reagents IR780 in tumor embolization therapy [37]. Thr rapidly forms clots in direct contact with blood because of strong coagulation, and this nonspecific embolism is extremely detrimental to normal tissues. The PCM consisted by 1-hexadecanol, oleic acid, l-*α*-Lecithin, and DSPE-PEG2000 has a wax sealing profile that prevents leakage of Thr during circulation. When applied 808 nm laser, IR780 triggers melting of the PCM to conduct tumor-specific embolization. Unlike the mentioned lipid vesicles, bicelles are phospholipid bilayer fragments with surfactants capped ends to stabilize edge. Traditionally, cholesterol as membrane stabilizer is a major component of most lipid-based carriers that imparts membrane fluidity but not suitable for bicelles, since it has a high critical micelle concentration (CMC) of cholesterol. Bicelles are unique two-dimensional materials, which brings special advantages for transdermal drug delivery since they are thin enough to pass through the narrow gaps between skin cells. Uchida et al. synthesized a new surfactant sodium cholate derivant (SC-C5) as edge stabilizer to form kinetically frozen bicelles at room temperature [38]. SC-C5 has extremely low CMC compared with traditional surfactants, which gives bicelles dilution tolerance and thermoresponsiveness (Figure 3(c)). The bicelles transform into micelles consisting of DPPC and SC-C5 upon heating to about 37°C. In addition to optimization based on the lipid itself, polymer doping is also a way to stabilize lipid-based nanoparticles. Lysolipid-based TSL (TTSL) is unstable in the body, and the blood DOX retention time is only 1 hour due to the inclusion of lysed lipids and lack of cholesterol. Mo et al. synthesized polymers involving poly(NIPAM-r-HPMA) and poly(HPMA-r-APMA) that proposed to be inserted into the lipid bilayer of TTSL by a postinsertion method [39] . The optimized TTSL can achieve nearly 70% DOX release in 1 minute at 42°C, while remaining stable at 37°C.

Thirdly, release on demand is a developing direction of lipid-based nanoparticle precise control. Reversible reaction is a dominant principle for multiple release. Wang et al. designed a lipopeptide based on leucine zipper as a thermosensitive on/off switch molecule that was mixed with phospholipids to form a hybrid liposome [40]. This recyclability is due to lipopeptide structure with a carbon chain and a leucine zipper that can form dimers by hydrophobic forces at physiological temperature and separate into single random coils above T_m_ 45°C (Figure 3(d)). Thus, performing intermittent heating can achieve a reversible drug release on demand.

### 2.2. Ultrasound Response for Lipid Permeability Regulation

Ultrasound is a mechanical wave of periodic vibration with frequency greater than human hearing range through a medium. At present, there are low-frequency ultrasound (LFUS) and high-frequency ultrasound (HFUS) used in medicine, and the frequency of LFUS is 20-100kHZ, while HFUS is greater than 1 MHz with higher energy [41]. Furthermore, high-intensity focused ultrasound (HIFU) involves focusing ultrasound energy into a small spot, similar to light, resulting in tissue ablation only at the focal point with minimal damage to surrounding and overlying tissue [42]. During propagation of ultrasonic waves through the medium, periodic extrusion and dispersion of the media molecules can produce cavitation effect which is widely used in the field of biomedical materials. The cavitation refers to the kinetic process, whereby tiny bubbles, present in a liquid, conduct multiple volume expansions, contractions, and eventual high-velocity disintegrations under periodic oscillations of the rarefied and compressed phases of ultrasound. These volume changes produce a series of mechanical, thermal, and chemical effects [43]. These can be used to achieve local delivery and improved distribution of drugs in tissues, especially combined with lipid-based nanoparticles since their excellent biocompatibility and fluidity. The complementary combination improves response efficiency, avoids stability about inefficient leakage, and enables on-demand release.

Firstly, the three effects of thermal, mechanical, and chemical triggered by ultrasound have a synergistic impact on drug release or treatment. Temperature response is essential for ultrasound response based on temperature-dependent phase transition. Ultrasound can be converted into thermal energy by friction between medium particles or interface as well as by absorption of medium. Compared to the thermal effect of AMF mentioned above, HIFU generates local concentrated heat in much shorter time (30 s) because of the focus. Thebault et al. utilized HIFU to locally heat thermosensitive supramagnetic liposomes loaded with combretastatin A4 phosphate at T_m_ [44]. Magnetic nanoparticles *γ*-Fe_2_O_3_ in this case are only used to enrich nanoparticles to tumor sites by external magnetic fields rather than heating.

**Figure 4:**
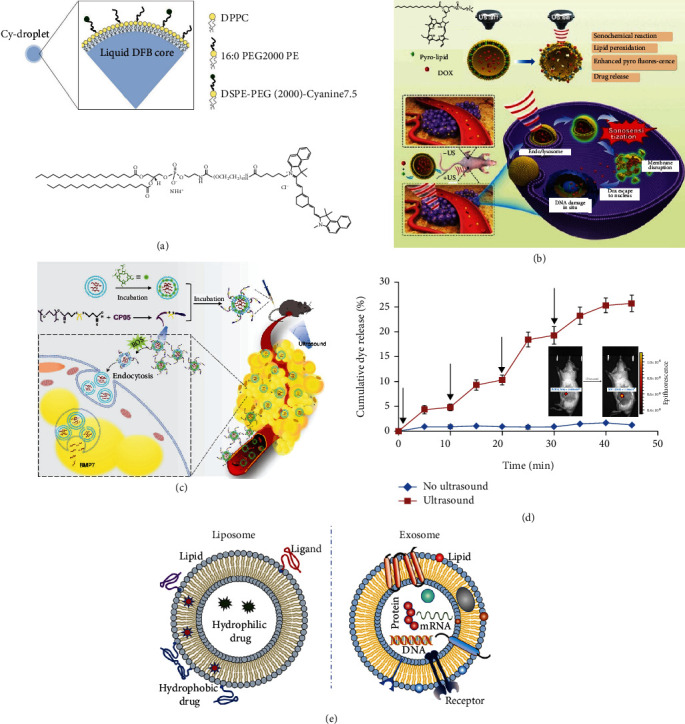
Ultrasound response for lipid permeability regulation. (a) Cy-droplet can conduct phase change by laser or ultrasound stimulation [47]; (b) porphyrin-conjugated liposome reduces required ultrasound intensity [49]; (c) PEG coat can be removed after ultrasound to enhance cell uptake [51]; (d) nerve block is controlled by insonation parameters, extent and intensity [55]; (e) schematic diagram of liposome and exosome [51] .

The mechanical effect of ultrasound can be achieved directly on liposomes attributed to formation of transient pores. Schroeder et al. designed a cisplatin release strategy through liposome under LFUS activation [45]. While the mechanical effect of ultrasound can be enhanced by combination with perfluorocarbon (PFC) droplets since their high volatile and gas solubility, also known as ultrasound-targeted microbubble destruction (UTMD) technique. Once exposed to ultrasound, the droplets form gaseous microbubbles and microjet flow during bubbles disintegrations to burst carrier boundary [43], which cause efficient release. Xuejiao Song et al. prepared perfluoro-15-crown-5-ether nanoemulsion with DPPC, DSPE-PEG and stabilizer albumin. The nanoemulsion can act as a shuttle to load oxygen at lung and burst in tumor by stimulation of LFUS. And the unloaded shuttle can cycle to lung for next transport [46]. Lin et al. developed a microbubble contrast agent so called Cy-droplet that has two alternative phase change mode, laser, and ultrasound stimulation. They encapsulated another perfluorocarbon (decafluorobutane, DFB) with DPPC, DSPE-PEG, and conjugate optical absorber Cyanine7.5 on the surface (Figure 4(a)) [47]. In addition to the above PFC-based UTMD, mechanical effect can also be combined with piezoelectric materials to achieve oxygen generation in situ rather than transport from other sites. Wang et al. constructed an ultrasmall DSPE-PEG2000 coated barium titanate (BTO) nanoparticle [48]. BTO is a piezoelectric material that generates an unbalanced charge state on surface under ultrasonic mechanical action. The excess charge cannot only react with water molecules directly to form O_2_ but also combine with H_2_O or O_2_ to produce reactive oxygen species (ROS) to kill tumor cells.

The chemical effects of ultrasound can also induce ROS that be used to enhance response release. Although it lasts only a short time (<1 *μ*s) for extreme microbubble, reactive chemicals, such as ROS, can also be produced by cavitation simultaneously, especially in the presence of acoustic sensitizers. Wan et al. prepared a porphyrin-conjugated lipid into liposome to deliver DOX [49]. The formulation can reduce the ultrasound intensity required for drug release from liposomes to 1.0 MHz. Increased Dox nuclear subcellular localization suggests release enhancement, which is associated with ROS production and liposome peroxidation (Figure 4(b)). The generation of free radicals not only contributes to load release but also to synergistic antitumor strategies based on sonodynamic therapy (SDT). Lin et al. combined UTMD and SDT. They developed a diagnostic 2,2′-azobis[2-(2-imidazolin-2-yl)propane]dihydrochloride-loaded (AIPH) liposome for sonodynamic therapy [50]. AIPH can produce both alkyl radicals and N_2_ under ultrasound. N_2_ bubble operates destruction of liposomes to release AIPH that generates radicals in a O_2_-independent manner, and on the other hand can act as contrast agent enhancer in ultrasound imaging.

Secondly, ultrasound can be a switch to adjust state of lipid-based nanoparticle to avoid premature elimination. It is not easy for exosomes to escape from the clearance by mononuclear phagocyte system (MPS) completely even after modification. Both liposomes and exosomes are lipid-based nanoparticles. They both have phospholipid bilayer as framework for drug delivery. Compared with liposomes, exosomes, as the main mediator of intercellular communication, have a large number of biogenic proteins, carbohydrate, and lipids in their membranes, and the core is usually encapsulated with nucleic acids or messenger substances. Compared with normal cells, exosomes have some special markers that contain high amounts of CD9, CD63, and CD81 and low *β*-actin (Figure 4(e)). Exosomes have many advantages such as weak immunogenicity, long cycle time, and strong targeting efficiency, but they are difficult for bulk preparation [51, 52] . PEGylation is a common strategy to enhance stability of nanoparticle by forming a hydrophilic corona to avoid MPS. But meanwhile it also reduces endocytosis by target cell. Guo et al. raised an ultrasound-triggered PEG-cleavable engineered exosome [53]. The invisible coat CP05 peptide-thioketone- polyethylene glycol (CP05-TK-mPEG) is anchored to the exosome marker CD63. Controlled removal of PEG can be achieved by destruction of thioketone TK by ROS generated by chlorin e6 under ultrasound irradiation (Figure 4(c)). Furthermore, it is an effective way to prevent drug leakage by conjugating drug on carrier shell rather than encapsulating it in the core as free form. Chen et al. used amphiphilic camptothecin-fluorouracil (CF) conjugate and other lipid materials to self-assemble into microbubbles encapsulated with PFC [54]. The CF microbubbles transform to CF nanoparticles after UTMD. At the same time, cellular uptake of transformed nanoparticles is significantly increased due to the acoustic pore effect that enhances the permeation of the cell membrane and the vascular system.

Thirdly, ultrasound-controlled release on demand is achieved by adjusting the applied parameters generally with less material specificity. Cao et al. prepared two kinds of nanodroplets with different stiffness, lipid-based nanodroplets and PLGA-based nanodroplets, for programmed drug release [55] . Based on acoustic pressure difference between soft-shell and hard-shell nanodroplets, they utilized programmable LIFU excite phase-change-dependent drug release by adjust parameter. Approximately 30% of DOX was released within the first 8 h without any stimulation. At the first LIFU (3 W, 3 min), the percentage of cumulative drug increased by 50%, and more than 20% of the drug release after second LIFU (8 W,3 min) radiation at 18 h. Alternatively, regulating ultrasound parameters unilaterally also allows control the amount of drug release. Nele et al. investigated cofactor-catalyzed gelation under ultrasound triggering [56]. In a certain condition with consistent parameters, frequency, power density for instance, total release amount depends on the exposure time. Applying 20 kHz ultrasound at 20% amplitude and 25% duty cycle, by adjusting the exposure time varying between 1 and 50 seconds. Finally, 92% is the maximum release amount of the total encapsulated calcium. Highly potent anesthetics are extremely suitable for on-demand release modes, such as tetrodotoxin that needs elaborate dose administration. The controlled drug release with high-precision perfectly fits the requirements of anesthetic blocking nerve including time point, intensity, and duration. Rwei et al. investigated extent and intensity of insonation, controlling the duration of the nerve block (Figure 4(d)) [57] .

### 2.3. Electromagnetic Wave Response for Lipid Conformation Stability

Traditionally, stimuli source of light-triggered lipid-based nanoparticles not only contains visible light but also extends to ultraviolet ray (UV) and near infrared (NIR) regions. Recently, investigations based on microwave (MW) and X-ray responses have been reported [15, 58–61]. Here, perhaps electromagnetic wave response is a more comprehensive perspective to summarize the development of light-triggered DDS. Electromagnetic waves are oscillating particle waves emitted by electric and magnetic fields derived in the same direction and perpendicular to each other in space. Visible light is a special type of electromagnetic wave with a narrow spectral window of 390-750 nm approximately. It is decreasing in order for wavelength of MW, NIR, visible, UV, and X-ray. Different wavelengths of light can cause chemical changes about carrier compounds, photoisomerization, photocleavage for instance, or be converted into other energy such as thermal energy [62]. According to the above photochemical properties, some examples will be presented below about improving carrier release efficiency, regulating carrier steady state and achieving on-demand release.

**Figure 5:**
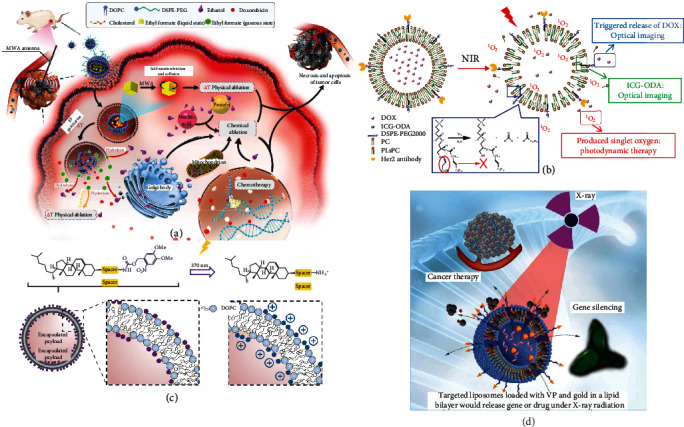
Electromagnetic wave response for lipid conformation stability. (a) MW converter EF blasts lipid shell by gasification to accelerate DOX release [57]; (b) PLsPC response to ROS that is generated by ICG after ultrasound [61]; (c) charge of liposome convers from neutral to cation through UV irradiation [62]; (d) X-ray induces singly linear oxygen by verteporfin to cause liposome response [59].

Firstly, release efficiency enhancement of the nanoparticles based on electromagnetic wave response depends on the development and optimization of sensitive switches. Generally, MW response is based transform of microwave energy into thermal energy through a converting agent, which is temperature response essentially. Ionic liquids are converting agents, such as sodium chloride. Zhou et al. prepared liposomes loading sodium chloride as effective thermo-seeds for microwave ablation of hepatocellular carcinoma [58]. But ion homeostasis is so critical for cellular homeostasis that this strategy lacks specificity and seriously endangers normal cells or tissues. Hou et al. proposed ethyl formate (EF) as a nonionic MW sensitizer to encapsulate in liposomes with DOX. EF not only converts electromagnetic energy to thermal like sodium chloride but also blasts lipid shell by EF gasification thus accelerates the release of DOX into the liver tumor (Figure 5(a)) [59]. Other sensitizers such as 1-butyl-3-methylimidazolium-l-lactate (BML) have the same effect. Xu et al. constructed a MW-responsive liposome by encapsulating DOX and BML into fucoidan-conjugated nanoparticles [60]. For hepatocellular carcinoma in orthotopic and in pulmonary metastases, the nanoparticle MW-assisted DOX-specific accumulation was equivalent to 10-fold higher doses of free DOX treatment.

There are two main principles of NIR response, of which applications based on photothermal effects have been described previously. And here, we mainly summarize photochemical-based response. There are few examples about NIR directly causes cleavage for lipid-based nanoparticles since only a few chromophores can be cleaved under NIR. But diverse chromophores can generate ROS under NIR to conduct photochemical-based response, which accompanies photodynamic therapy. Li et al. embedded hydrophobically modified indocyanine green (ICG) into liposomes containing PLsPC phospholipids (Figure 5(b)) [63] . And the light sensitive liposome can be destroyed by ROS that generated through ICG under NIR.

Compared with NIR, UV has higher reaction activity to photosensitizer but poor penetrativity. Arias-Alpizar et al. described a light-triggered switch in charge conversion on the liposome surface. Under 240 nm UV irradiation, liposomes rapidly adsorb to endothelial cells and are taken up by endothelial cells and/or phagocytosed by macrophages in the blood because of the conversion from neutral to cation (Figure 5(c)) [64]. Moreover, a variety of molecular switches have been developed, azobenzenes, spiropyrans, hexaarylbiimidazoles, and hydrazones for instance, due to UV being prone to bond isomerization [65]. But UV is limited for its poor penetration, which will be addressed by the following upconversion strategy.

X-ray has super penetrating power since it has high energy. Therefore, X-ray triggered materials can almost completely solve the problem of deep tissue response. Deng et al. used X-ray to destroy stability of liposomes based on photodynamic response [61]. The 6 MeV X-ray radiation induces the production of singly linear oxygen by verteporfin, which destabilizes the liposome membrane and leads to the release of cargo from lumen (Figure 5(d)). It is worth noting that NIR and X-ray both reach deep tissue on different principles. NIR penetrates into tissue by thermal radiation with a depth of centimeter scale, while X-ray transmits organism totally since it has high energy.

Secondly, photocatalytic degradation can be used as a switch to regulate the steady state of nanoparticles. There is a paradox for nanoparticles between stable long circulation of and efficient cellular uptake. While improving the uptake of nanoparticles by target cells, it also enhances the probability of being cleared by MPS or reticuloendothelial system (RES), as highly specific uptake is difficult to achieve. Here are two examples about light switch regulating stable state of nanoparticle. A work involves light-controlled cleavage of charge neutralizer group. Cell-penetrating peptides (CPP) enhance cellular uptake of nanoparticles. In order to avoid nonspecific elimination of CPP-modified liposomes in circulatory, Yang et al. attempted to neutralize the positive charge of CPP based on o-nitrobenzyl derivatives, which causes CPP deactivation temporally [66]. Reactivation of CPP is realized by NIR irradiation at tumor site, which can cleave the derivatives. Another work involves light-controlled degradation of cell membranes. Surface coating of nanoparticles through natural cell membranes can improve stability and prolong circulation time. However, the cell membrane coating cannot be effectively degraded in the tumor microenvironment, which greatly affects the drug release from nanocarriers. Rao et al. used the photocatalytic activity of titanium dioxide (TiO_2_) to control the degradation of erythrocyte membrane coatings on nanoparticle surfaces [67]. TiO_2_ as a semiconductor can form a redox system of electron-hole pairs on surface under UV, which in turn degrades the lipids and proteins of the red blood cell membrane.

Thirdly, reversible photochemical reaction is dominant basis of photo-controlled release on-demand. When the radiation is applied, sensitive molecule is in “on mode” while the molecule changes back to its original conformation when the excitation source is withdrawn, in another word, “off mode”. Tong et al. launched a photoswitchable nanoparticle that enables repetitive dosing from a single administration [68]. They developed a photo-switchable molecule spiropyran (SP) that consists of a nitrobenzopyran and an indoline moiety with orthogonal orientation. SP conducts isomerization under UV irradiation to a zwitterionic form. This hydrophilic change drives reformation of lipids physical assembly properties. Importantly, the unstable zwitterionic form undergoes spontaneous ring-closing back to SP in the dark. However, in-depth biological application of reversible isomerization is limited by permeability of UV. Upconversion nanoparticles (UCNPs) can convert NIR to UV/Vis emission, which can solve this problem. Upconversion luminescence is a phenomenon in which two or more low-energy photons are absorbed accompanied with one high-energy photon which is emitted. As same with SP, azo group can realize an “on and off state” release kinetics of reproducible multiple release cycles. Yao et al. combined the tissue permeability of NIR and reversible isomerization of azo group based on UV to produce continuous rotational inversion motion of the liposome membrane [69]. Emitted region causes isomerization of the azo group of the lipid framework, resulting in drug release.

## 3. Physiological Environment Dependent Lipid Vehicle for Smart Delivery

### 3.1. pH Dependent Lipid-Based Drug Delivery

**Figure 6:**
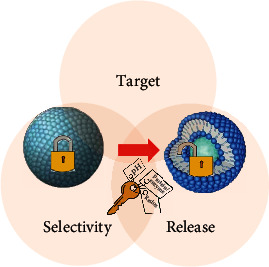
Three main aspects of physiological signal-activated lipid-based nanoparticles.

Biochemical reactions occur in the human body all the time, and these processes are often accompanied by changes in pH (Figure 6). Under normal physiological conditions in the human body, the pH of plasma and tissues exhibits neutrality maintained at about 7.4. In contrast, the extracellular microenvironment of tumors exhibits a weak acidic pH between 6.5-7.2. When it comes to intracellular tumor, the pH decreases further, such as only 4.0-6.0 in lysosomes and endosomes, and these pH changes in different parts of the body can bring new ideas for the design of drug delivery [70, 71]. For example, by taking advantage of the lower pH of tumor tissues compared to normal tissues, drugs can be delivered deeper into the tumor to achieve targeted delivery at the tumor site and accumulate to produce better therapeutic effects [72, 73].

**Figure 7:**
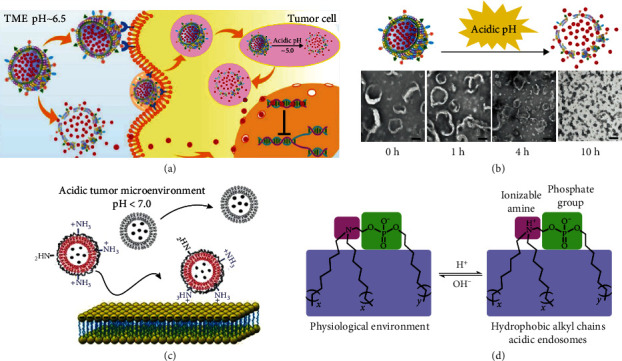
pH dependent lipid-based drug delivery. (a) platesome pH-response process; (b) platesome in vitro pH-responsive cleavage process [75]; (c) exposure to positive charges in the tumor microenvironment at pH < 7.0 promotes cellular uptake [76]; (d) iPhos lipids consist of an ionizable amine phospholipid head and three hydrophobic alkyl chain tails [81].

Currently, there are two main ways to deliver drugs in response to the acidic pH of tumor microenvironment, one is to use pH-sensitive chemical bonds to construct carriers, such as hydrazone, ester, and ligand bonds, which remain stable at physiological pH and can break under acidic conditions to release drugs [1, 74, 75]; the other is to design carriers with materials containing protonated or deprotonated groups, such as amino, hydroxyl, carboxyl, etc., which can produce a shift between hydrophilic and hydrophobic under acidic conditions or change the charge properties of the carrier to depolymerize the carrier then release the drug or enhance drug uptake and thus improve the therapeutic effect [76]. The mentioned is still the conventional approach to confer pH-responsive function to lipid materials, such as Mishra et al. formed lipid nanoparticles (DOX-B6-SA-NP) by linking vitamin B6 to stearic acid and loaded with DOX, which can enter tumor cells through vitamin transport receptors. The pKa = 5.6 of vitamin B6 showed positive charge upon entry into tumor cell endosomes (pH = 5.0) allowing both endosomal escape and drug release [77]. Liu et al. introduced a bionic concept based on conventional pH-responsive liposomes loaded with DOX by fusing them with platelet membranes to form platesome, which can be stealthy in vivo and with active targeting to tumors, thus enabling efficient treatment of tumors [78]. It can release drugs at low pH in response to the tumor microenvironment and endosomes, and in in vitro tests, it can release drugs completely at pH = 5.0 within 4 h (Figures 7(a) and 7(b)). Overall, the general pH response serves mostly the purpose of endosome escape and drug release, with some other studies currently available to address cellular uptake and drug loading. Yan et al. designed a drug delivery system based on Glycol Chitosan (GC)–Coated Liposomes; they used HSPC, cholesterol, and DSPE-PEG2000-COOH as the basic material for the synthesis of liposomes. GC can react with the carboxyl group on DSPE-PEG2000-COOH and modify the liposome surface in a chemically bonded manner [79]. The successfully modified liposomes have an increased particle size and exposed amino groups on the surface. When reaching the tumor site through the enhanced permeability and retention (EPR) effect, the amino groups are protonated due to the decrease in pH resulting in a negative to positive charge shift of the liposomes (Figure 7(c)). Such a charge shift allows liposomes to interact better with negatively charged cell membranes, thereby enhancing drug uptake by tumor cells.

In mRNA delivery, nucleic acids need to go to the cytoplasm to function, so endosomal escape is the basis for effective drug delivery of nucleic acids, and there are few carriers that can achieve this function even the Dood and Drug Administration-approved DLin-MC3-DMA LNP can only achieve a very small portion of successful delivery [80, 81]. Phospholipids are key components of biological membranes and organelles found throughout nature, and therefore can be added to carriers to break the lysosomal barrier [82, 83]. Currently commonly used phospholipids, such as DSPC and DOPE, have relatively constant structures that are difficult to further modify, and thus are subject to further development. Inspired by cationic lipids, Liu et al. summarized their advantages; the ionizable amine contained in the structure can achieve from neutral physiological environment to acidic endosomes; exposure of positive charge by pH change can rupture the endosomal membrane, and secondly, the attachment of more than two alkyl chains at the head of the amphiphiles can make the spatial structure more prone to cone formation, thus causing a stronger tendency of membrane phase transition (Figure 7(d)) [84]. To achieve these functions, they designed a total of 572 iPhos lipids, all containing one tertiary amine, one phosphate group and three alkyl chains, and used this lipid library to screen the most promising agents for gene editing applications, among which the LNP formed by iPhos 9A1P9 is 40-965 times more efficient for gene delivery and editing in vivo compared to the conventional LNP; it can be more specifically enriched in the liver or lung in the presence of different helper lipids. In addition, it is worth noting that this improved efficiency is also dependent on the specific transfection of extrahepatic organs by this agent, as we will continue to discuss below.

### 3.2. ROS/GSH Activated Sulfur-Based Lipid Cleavage for Drug Release

In normal cells, the redox state is relatively balanced, but in tumor cells, this redox balance is disrupted and there is an overall state of oxidative stress, i.e., an imbalance between reactive oxygen species (ROS) production and resistance to oxidative defenses [85–87]. Thus, in tumor cells, both ROS and reduced glutathione (GSH) are in a state of high expression, reflecting 100-fold and 7-10-fold higher than in normal cells, respectively [88, 89].

**Figure 8:**
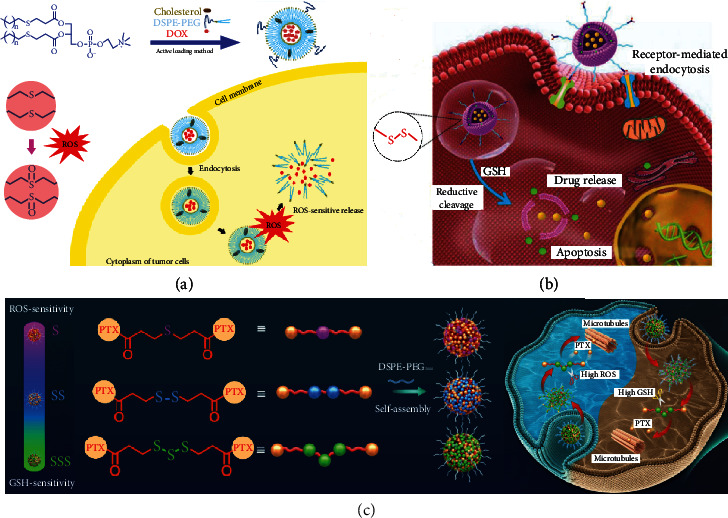
ROS/GSH activated sulfur-based lipid cleavage for drug release. (a) Thioether-containing lipid carrier for ROS-responsive drug release [91]; (b) Disulfide-containing lipid carrier for GSH-responsive drug release [92]; (c) Trisulfide-containing lipid carrier for ROS and GSH dual-responsive drug release [100].

In the face of tumor cells, it is easy to think of using high local ROS or GSH to accomplish targeted delivery and controlled release of drugs. A variety of chemical bonds have been reported to achieve the response to ROS, such as thioether, selenium, thioketal, etc., and thioether is the most common and simple one [90–93]. Li et al. synthesized a thioether-containing lipid material (S18-PC) like DSPC and used it to synthesize liposomes loaded with DOX, which can be maintained stable in normal environment and can be released at 10 mM concentration of hydrogen peroxide (Figure 8(a)). And in this concentration, it can achieve more than 80% of the drug release in 24 h [94]. They verified its effectiveness in treating the 4 T1 mouse model. The most used chemical bond for GSH response is disulfide or diselenide. Wang et al. prepared a GSH-responsive liposome with surface modification of liver tumor stem cell targeting ligands, which GSH-responsive portion consisted of lipid materials containing disulfide bonds (DSPE-SS-PEG2000) and loaded with both salinomycin and DOX for targeted synergistic therapy of hepatocellular carcinoma to address the current problem of high drug resistance and recurrence of this disease (Figure 8(b)) [95]. Huang et al. first prepared biocompatible and stable Fenton catalyst GA-Fe (II) nanocomposites by mixing Fe (II) with gallic acid (GA). Subsequently, GA-Fe (II) was cis-encapsulated in liposomes modified with cisplatin prodrugs and mitochondrial targeting groups Triphenylphosphorus (TPP) to construct multifunctional composite nanocatalysts Pt/GF@Lipo-TPP, in which cisplatin prodrugs could be used to provide additional hydrogen peroxide for the Fenton reaction in response to GSH release in tumor cells and could produce DNA damage for synergistic treatment [96].

In fact, it can be seen from the previous paper that by inserting different numbers of sulfur into the lipid material, the response sensitivity of the lipid material will be changed accordingly, such as thioether responds to ROS, while disulfide bond has a certain degree of double response, which is mainly applied to respond to GSH. In addition, disulfide bond is also a kind of dynamic covalent bond that exists in large quantities in proteins. The introduction of disulfide bonds into the structure of prodrugs cannot only play a role in enhancing the structural flexibility of the prodrug molecule but also confer the role of prodrugs to respond to redox signals in the tumor microenvironment [97, 98]. Disulfide bonds have long been used as the “gold standard” for the design of prodrug self-assembly nanodelivery systems [86, 99]. Based on this, Yang et al. challenged this “gold standard” by inserting a trisulfide bond with higher sensitivity to GSH into the homodimer of DOX, and prepared a nanoformulation with high drug loading capacity, high self-assembly stability, and high tumor selectivity, in which DOX is linked to the trisulfide bond by an amide bond [100]. However, under physiological conditions, the amide bond is difficult to hydrolyze, so the DOX acts through DOX-SH [101, 102]. However, this is not universal for drugs that need to occupy the active site for modification, such as paclitaxel-like drugs commonly used in clinical practice. Therefore, Yang et al. again chose paclitaxel as a model drug to further investigate the assembly and release of the prodrug in depth and validated the sensitivity of the prodrug for differentiating the response of normal and tumor cells in in vivo and in vitro studies [103]. Moreover, they also discovered and reported for the first time that trisulfide bonds are also capable of oxidation reactions (Figure 8(c)). This provides another option for the future direction of progress of redox-responsive lipids, whose sensitivity can be further improved by introducing trisulfide bonds, and when comodification with the prodrug is required, the physicochemical properties of the prototype drug should also be fully considered. In the future, it could also be combined with imaging techniques to visualize diseases with microenvironmental redox alterations by linking fluorescent probes to more sensitive response units, allowing the detection of disease processes at the cellular level.

### 3.3. Lipid-Based Drug Delivery Strategies Triggered by Disease-Related Enzymes

Enzymes are an essential component of chemical reactions in living organisms, which not only ensure that the reaction can be specific in a complex physiological environment but also accelerate this reaction and obtain the desired product more quickly. Enzymes are expressed at specific sites in the organism and will exhibit different activities in different tissues. In specific diseases, high concentrations of specific enzymes are always detected at the site of the lesion, and indeed, the disease arises from dysregulation of the expression of one or more enzymes [104]. In the tumor microenvironment, the rapid growth of tumors cannot be achieved without the assistance of some nontumor cells, which highly express enzymes that facilitate tumor invasion and metastasis within the microenvironment [105]. Therefore, we can engineer carriers by exploiting the sites of action of these enzymes to enhance the release of drugs at the target sites and reduce off-target effects.

**Figure 9:**
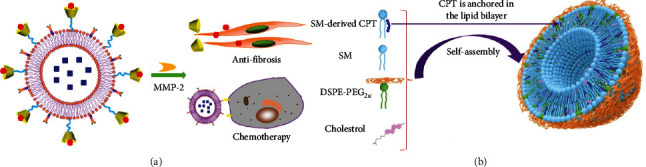
Lipid-based enzyme-responsive drug delivery. (a) MMP-2 responsive functional liposomes inhibit tumor mesenchyme thereby enhancing chemotherapeutic drug perfusion [106]; (b) Functionalized sphingomyelin self-assembles to form camptothesome [114].

Matrix metalloproteinases (MMPs) are calcium ion-dependent enzymes that degrade the extracellular matrix. They can disrupt histological barriers during tumor invasion, neoangiogenesis, and metastasis [106]. They are highly expressed in the tumor microenvironment compared to normal tissue, and MMPs exist in multiple isoforms and are a persistent and attractive target for cancer therapy [107]. Nie et al. linked *β*-cyclodextrin encapsulated with pirfenidone (PFD) and liposomes encapsulated with gemcitabine (GEM) via an MMP-2 responsive peptide, while the surface of the liposomes was modified with RGD targeting peptide (Figure 9(a)) [108]. When used for the treatment of pancreatic cancer, the peptide can be broken by MMP-2 response to first release PFD to alleviate the interstitial fibrosis of pancreatic cancer tumor microenvironment, after which the GEM-encapsulated targeting liposomes can better enter the deep tumor layer to act, thus effectively improving the therapeutic effect of pancreatic cancer. In addition, Deng et al. developed a DOX liposome (PEG-FA-Lip) that can be triggered by MMP-2 as a targeting “switch”, with the liposome surface modified with both folic acid (FA), and a PEG chain that can respond to MMP-2 breakage [109]. When used in mice with breast cancer, PEG-FA-Lip cleaves PEG chains in response to MMP-2 in the tumor microenvironment, exposing FA to target 4 T1 cells and M2 tumor-associated macrophages (M2-TAM) at the tumor site.

Phospholipase A2 (PLA2) is an enzyme that is widely distributed in mammalian cells and has the function of cleaving fatty acids. Studies have shown that PLA2 is highly expressed in advanced cancers of the digestive organs, such as gastric [110], pancreatic [111], and prostate cancers [112]. Since PLA2 needs to act on negatively charged lipid surfaces when performing its hydrolytic function, DSPG and DSPE can be preferred for application in phospholipase-sensitive lipid materials. However, this process of releasing drugs solely using lipid materials in response to enzymatic depolymerization lacks controllability, and the stability of lipid carriers at normal sites requires constant attention, so the lipid ratios must be carefully designed [113] .

The toxic side effects due to off-target effects can be effectively reduced by designing enzyme-responsive prodrugs [114]. Pedersen et al. prepared PLA2-responsive lipid prodrugs by modifying the anionic lipid DSPG with all-trans retinoic acid (ATRA) attached to the hydrophobic chain on one side by introducing ester bonds and assembling them with DPPC to form liposomes [115]. The ATRA-based lipid prodrug completely released ATRA in the presence of PLA2 within 24 h and exhibited cytotoxicity. Lu et al. developed a sphingomyelin- (SM-) derived camptothecin (CPT) nanovesicle therapeutic platform by linking and self-assembling CPT with SM through chemical bonds with different responsiveness (ester bond, disulfide bond, and glycine) to form a camptothesome, through which the chemical linkage can effectively improve the lactone stability of CPT and avoid systemic toxicity (Figure 9(b)) [116]. They used the camptothesome to bind chemotherapeutic drugs or immunosuppressants, thus effectively inhibiting or curing multiple cancer models.

### 3.4. Protein Corona Modulated Lipid Vehicle Endogenous Target In Vivo

The responsive delivery using changes in pH, redox, and enzymes conditions at the site of the disease, as described in the previous three sections, is still limited by the fact that these responsiveness mostly originate from a microenvironmental or intracellular change, yet the basis of the disease mostly originates in an organ, and most of the current intravenous procedures for drug delivery enrich it in the liver, so a better solution is needed for drug delivery to extrahepatic tissues, where only reaching the target organ first is more effective in reducing peripheral side effects and better drug utilization [117, 118]. Endogenous targeting is one of the goals of current scientific research and the future clinical field, that is, instead of using specific molecules to modify the carriers, the structure and ratio of the components of the carriers are adjusted so that it can selectively deliver proteins or small molecules to an organ or cell in the body [119, 120].

**Figure 10:**
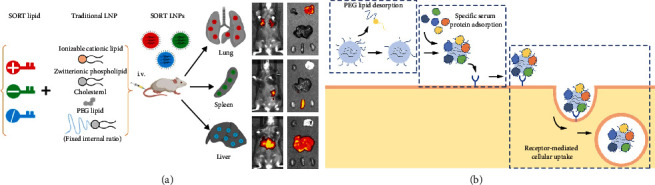
Protein corona mediates SORT LNP tissue-specific delivery. (a) SORT LNP achieves tissue-specific targeting [121]; (b) SORT LNP is an endogenous targeting mediated through the principle of protein corona adsorption [122].

While conventional LNP generally consists of four lipid components: ionizable cationic lipids, amphiphilic phospholipids, cholesterol, and poly (ethylene glycol) (PEG) lipids [121, 122], Cheng et al. analyzed that by adding a fifth lipid (SORT) to the components of conventional LNP and by adjusting the ratio of different SORT lipid components, they could change the organ targeting specificity of LNP in vivo and could enable the delivery of mRNA to organs other than the liver (Figure 10(a)) [123]. More importantly, this SORT-LNP can be extended to a variety of extrahepatic organs and tissues, enabling mRNA delivery to lung, kidney, spleen, and even epithelial cells and immune cells. They also considered that gene delivery requires endosomal escape to function in the cytoplasm, so they designed an iPhos phospholipid consisting of an ionizable amine head and three alkyl chains as a tail, based on the ability to specifically deliver to extrahepatic tissues, from which the LNP can effectively achieve organ-selective delivery and endosomal escape in target cells, with an in vivo efficiency of up to 965-fold compared to conventional LNP [84].

Following this, Dilliard et al. further analyzed the specific mechanism by which SORT-LNP breaks through the hepatic accumulation limit to achieve organ-tissue specific targeting [124]. This helped to drive the optimization of SORT-LNP for therapeutic applications in tissues and organs such as lung, liver, and spleen, greatly expanding the scope of mRNA vaccines and drugs as well as CRISPR gene editing therapies. This study found that the chemistry of the added SORT molecule controlled the organ-level biodistribution, acid dissociation constant (pKa), and serum protein adsorption of SORT-LNP. Specifically, PEGylated lipids dissociate from the LNP surface and expose SORT molecules, different serum proteins recognize the exposed SORT molecules and adsorb to the LNP surface, and surface-adsorbed proteins interact with cell-expressed cognate receptors in target organs to facilitate mRNA delivery to these tissues (Figure 10(b)). These findings establish a critical link between the molecular composition of SORT-LNP and its unique and precise organ-targeting properties and suggest that recruitment of specific proteins to the LNP surface can enable targeted delivery outside the liver. The results of this study suggest that endogenous targeting, that is, tuning the molecular composition of nanoparticles to bind to specific proteins in the serum so that they can be delivered to the target site may be an effective strategy for achieving extrahepatic delivery of nanocarriers.

## 4. Summary and Perspective

In summary, diverse lipid derivatives enable lipid-based nanoparticle can be activated in physical and physiological stimulation (Table 1). Thus, lipid-based smart nanoparticles can respond to conditions in a predesigned manner, which benefits for efficiency enhancement or side effect avoiding reflected in spatio-temporal control and pharmacokinetic behavior. Comparing the physical and physiological responses, they each have advantages and disadvantages. Thermal response is proven and available in terms of materials. But it needs photo-thermal, acoustic-thermal to achieve energy conversion since direct temperature increase is difficult to achieve in therapy. Physical response, ultrasound, and electromagnetic wave for instance, can achieve good targeting but has limited penetration in deep tissue. Compared to responses that require physical conditions to trigger, responses based on altered physiological conditions can be designed to eliminate the need for in vitro manipulation, and there is no concern that the response signal will not reach deeper tissues. However, the disadvantage is that physiological conditions are not unique to a particular site compared to the physical signal of an absolute stimulus, and therefore, how to avoid peripheral toxicity during drug delivery is the current focus of physiological conditioned responses.

From the perspective of physical conditions, thermal response represented by temperature-sensitive liposomes has developed mature materials, lysolipids, already in next step clinical trials. TSL is the closest to clinical practice among the intelligent response materials. However, data from clinical trials do not support significant efficacy in TSL with combination of radiofrequency ablation, which induce drug release by directly heat activation. Optimized protocol of clinical trial should be carried out, including ablation time and primary endpoint selection. On the other hand, a variety of energies can be converted into thermal, indirectly causing drug release, for instance, light, alternating magnetic fields, ultrasound, and microwave. However, these indirect responses are far away from clinic application since low release efficiency and high damage to normal tissues in a complex organic environment. To address these issues, intelligent drug delivery system needs to balance delivery targeting with controlled responsiveness, as if a sensitive switch was wired for navigation. Although it has been reported that many targeting molecules were modified on the surface of nanoparticles, the targeting effect comes up to expectations especially at systemic organ level. One of the reasons is that an underlying problem has not been solved that efficient nanoparticle penetration through the circulating-tumor barrier.

Other response strategies have their own specific characteristics. Response kinetics of the carrier based external signals, especially for ultrasound, can be adjusted by certain parameters such as applying position, start moment, duration, and signal intensity. Ultrasound is a more mature technology used in clinical practice, and the standards for various parameters have a more complete system. Given ultrasound has multiplied in clinical practice; nanoparticles based on ultrasound response are considered to have a high potential to become the first choice for noninvasive delivery. But spatial resolution needs to be improved because the signal from a single exogenous radiation is difficult to locate precisely for human body in three dimensions. It may be a helpful strategy that precise localization to a lesion through multiple sources of radiation. And diverse photosensitizers allow for low chemical barrier reactions in a relatively stable manner. Thus, another tendency of lipid-based smart nanoparticles is a combination of various response strategies, not just staying simple addition of existing reports but developing new response strategies in conjunction with proven methods, X-ray response in synergy with radiotherapy or chemotherapy for instance. Lastly, on-demand release field requires acceleration to precisely control response dynamics, whether based on material design or parametric protocol optimization, which is benefit for on/off control or multiple release of drugs.

From the perspective of physiological conditions, some diseases can lead to the disruption of various homeostasis in the body, such as pH balance, redox balance, enzymes balance, etc. In the current research, materials designed for changes in physiological conditions have been very widely used, and in the course of therapy, these materials can be further modified on this basis (e.g. surface modified antibodies, organelle-targeting ligands, fusion with cell or platelet membranes, etc.) to enhance their targeting based on the specific environment generated in response, thus enhancing cellular uptake or release in the local microenvironment to achieve the desired effect. From a therapeutic point of view, whether based on physical stimulus response in vitro or physiological conditions in vivo, the carrier needs to first reach a specific tissue or organ and then exerts its effects on enhanced cellular uptake, receptor ligand-mediated transport, and microenvironmental response release. Therefore, enrichment of carriers in a particular tissue or organ is the most important direction for future research. Currently, the problem of drug delivery in extrahepatic tissues has been solved by modifying phospholipid materials to form LNPs that can adsorb proteins in serum and use protein crowns to accomplish endogenous targeting. However, these lipids with different responsiveness are still a long way away from practical application and clinical approach. The first is the storage of these lipids, as they have more sensitive chemical bonds than other lipids, which may cause them to be unstable in storage environments. Secondly, the use of these lipids as drug carriers, there are few reports on the safety of their long-term use and dosing issues, such as drug leakage from nonsite of action and organ toxicity due to high redox microenvironment in the liver. Third, the alteration of their local environment may not be significant in some diseases, which may result in current delivery strategies still not meeting the demands. Therefore, more research is needed to prove the feasibility of this series of hypotheses on the targeting, stability, and safety of lipid carriers.

It is hoped that in future research, all the above problems can be solved, and responsive lipid nanocarriers become a more powerful delivery tool for us, so that our nanocarriers can always reach their destinations in complex physiological structures shaped like mazes and use the unique conditions as keys or remoter to accomplish our demands.

## Figures and Tables

**Table 1:** Summary of lipid and cholesterol derivatives for lipid-based nanoparticles.

Chemical formula	Molecular structure	Application	Reference
1,2-dipalmitoyl-sn-glycero-3-phosphocholine (DPPC)	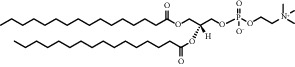	Commonly lipid materials to prepare lipid-based nanoparticle	[20, 21, 25, 30, 36, 38, 43, 47, 58]
Hydrogenated soy *sn* -glycero-3 -phosphocholine (HSPC)	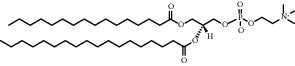	Has been used in DOXIL® and AmBisome®	[20]
1, 2-diheptadecanoyl-sn-glycero-3-phosphocholine (DHPC)	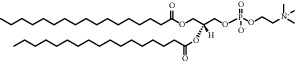	Hard to be oxidizedpreserve native protein structure	[25]
1-palmitoyl-2- hydroxy- sn -glycero-3-phosphocholine (MSPC)	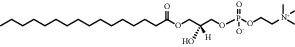	Temperature sensitive, easy to phase change	[21–23]
1,2-distearoyl-sn-glycero-3-phosphoethanolamine-N-polyethylene glycol 2000 (DSPE-PEG2000) (DSPE-MPEG)	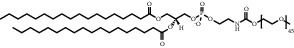	Commonly lipid materials to extend circulation time	[21, 25, 29, 30, 36, 37, 43, 47, 48, 58]
Cationic lipid MVL 5	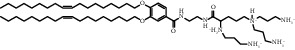	Multivalent cationic lipid for siRNA delivery	[25]
1, 2-dioleoyl-3-trimethylammoniumpropane (DOTAP)	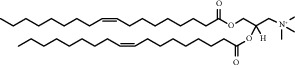	Commonly used cationic lipids for delivery of nucleic acids	[29]
Dioleoyl-phosphatidylethanol-amine (DOPE)	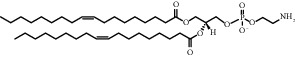	Commonly used helper lipid with DOTAP	[29]
1,2-dipalmitoyl-sn-glycero-3-phospho-(1′-rac-glycerol) (DPPG)	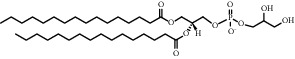	Commonly used lipids for TSL	[30]
Sodium cholate derivant (SC-C5)	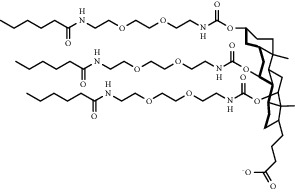	Edge stabilizer with extremely low CMC	[38]
Porphyrin-conjugated lipid (pyro-lipid)	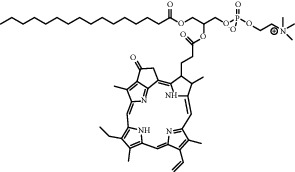	Ultrasound sensitization to generate ROS	[49]
CP05 peptide-thioketone- polyethylene glycol (CP05-TK-mPEG)	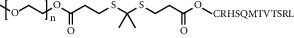	ROS response since thioketone	[51]
PLsPC phospholipids	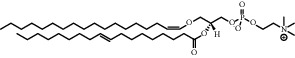	ROS response since oxidative breakage of ethylene ether bond	[61]
Vitamin B6-stearic acid(Vit-B6-SA)	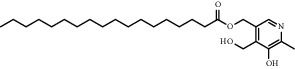	Acidic conditions can ionize and expose positive charges to enhance uptake	[75]
DSPE-PEOz	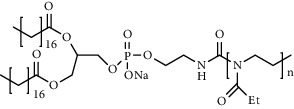	Breakable at low pH conditions	[76]
Distearoyl phosphatidylcholine(DSPC)	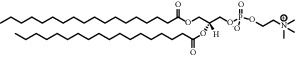	Helper lipid	[82]
9A1P9	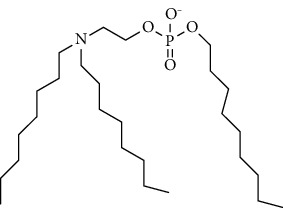	Endogenous targeting can be achieved by adjusting the doping ratio	[82]
S-18-PC	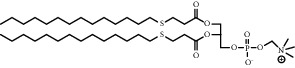	Change from amphiphilic to hydrophilic after responding to ROS	[92]
DSPE-SS-PEG2000	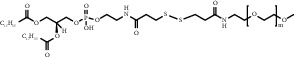	Disulfide bond breakage after response to GSH	[93]
DOX-(S)_n_-DOX	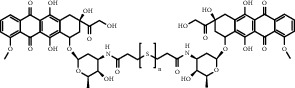	Depending on the introduction of sulfur atoms in the structure (1-3), different response releases of DOX can be designed	[98]
PTX-(S)_n_-PTX	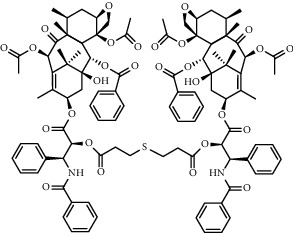	Depending on the introduction of sulfur atoms in the structure (1-3), different response releases of PTX can be designed	[101]
DSPE-PEG(3400)-pep-CD, CSSSGPLGIAGQSSS	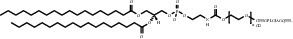	Release of *β*-cyclodextrin by peptide breakage in response to MMP-2	[106]
All-trans retinoic acid (ATRA) prodrug	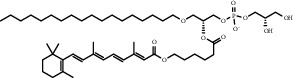	Activation of ATRA prodrugs after response to PLA2	[113]
Camptothecin sphingomyelin derivatives	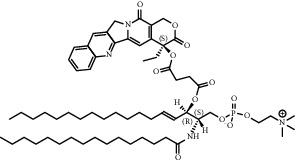	Multiple responses can be designed to activate camptothecin prodrugs	[114]
18PA	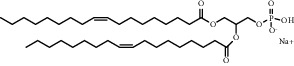	Anionic spleen-selective lipids	[122]
DDAB	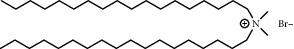	Cationic spleen/lung-selective lipids	[121]
EPC	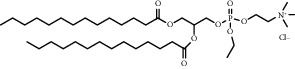	Cationic spleen/lung-selective lipids	[121]
14PA	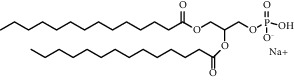	Anionic spleen-selective lipids	[121]
18BMP	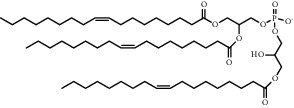	Anionic spleen-selective lipids	[121]
DODAP	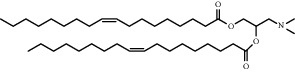	Neutral liver selective lipids	[122]
C12-200	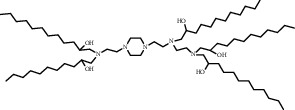	Neutral liver selective lipids	[121]
